# Isolated abducens nerve palsy associated with coronavirus disease: an 8-month follow-up

**DOI:** 10.5935/0004-2749.20220063

**Published:** 2025-08-22

**Authors:** Analine Lins de Medeiros, Thayze Martins, Marisa Kattah, Ana Karine A. Soares, Liana O. Ventura, Camila V. Ventura, Eveline Barros

**Affiliations:** 1 Department of Ophthalmology, Fundação Altino Ventura, Recife, PE, Brazil; 2 Department of Ophthalmology, Hospital de Olhos de Pernambuco, Recife, PE, Brazil; 3 Department of Scientific Research, Fundação Altino Ventura, Recife, PE, Brazil

**Keywords:** Coronavirus infections, Abducens nerve diseases, Strabismus, SARS virus, Ocular motility disorders, Humans, Case reports, Infecções por coronavirus, Doenças do nervo abducente, Estrabismo, Vírus da SARS, Transtornos da motilidade ocular, Humanos, Relatos de casos

## Abstract

We report the case of a previously healthy 48-year-old man who developed an isolated abducens nerve palsy 18 days after presenting with coronavirus disease (COVID-19) confirmed by reverse transcriptase polymerase chain reaction. His main complaint at arrival was double vision. Ocular examination revealed a sixth cranial nerve palsy in the left eye. The incomitant esotropia at arrival was 30 prism diopters. Abduction was markedly limited, while adduction was normal in the left eye. The patient underwent complete clinical, neurological, and neuroimaging investigations, including cerebrospinal fluid sample analysis to rule out infectious causes. A conservative approach with orthoptic therapy and Fresnel prism was opted. Eight months after the onset of COVID-19, regression of the strabismus was observed, and the patient reported complete recovery of the diplopia. This case suggests that isolated abducens nerve palsy caused by severe acute respiratory syndrome coronavirus 2 infection may improve with a conservative approach.

## Abstract (pt)

Reportamos o caso de homem previamente hígido, 48 anos, com paralisia isolada do nervo abducente 18 dias após infecção pelo novo coronavírus (COVID-19) confirmada por rea ção cadeia polimerase de transcriptase reversa. A principal queixa do paciente na admissão era diplopia. O exame ocular revelou paralisia do sexto nervo craniano do olho esquerdo. Esotropia incomitante no exame inicial media 30 dioptrias prismáticas. Abdução estava limitada com adução completa no olho esquerdo. O paciente foi submetido a investigação clínica e neurológica com exame de neuroimagem, incluindo análise de amostra do líquido cefalorraquidiano para descartar causas infecciosas. Optou-se por abordagem conservadora com terapia ortóptica e prisma de Fresnel. Oito meses após a infecção pelo COVID-19, o paciente evoluiu com regressão do estrabismo e informou recuperação completa do quadro. Este relato sugere que paralisia isolada do nervo abducente causada por SARS-CoV-2 pode melhorar com abordagem conservadora.

## INTRODUCTION

Severe acute respiratory syndrome coronavirus 2 (SARS-CoV-2) is a single-stranded RNA betacoronavirus that causes the coronavirus disease (COVID-19). The first case of the infection was reported in Wuhan, China in 2019, and rapidly reached a pandemic scenario with high numbers of cases and deaths worldwide^(1)^.

The most common symptoms at disease onset are fever, cough, fatigue, sputum production, headache, hemoptysis, diarrhea, dyspnea, and lymphopenia^(2)^. Other peripheral nervous system manifestations such as taste, smell, or vision impairment, and neuropathy were also related^(2)^. Ocular signs and symptoms are frequently observed in severe cases. The main manifestation is conjunctivitis characterized by epiphora, conjunctival hyperemia, and chemosis^(1)^.

The SARS-CoV-2 has neurotropic and neuroinvasive capabilities^(3)^. Bilateral optic neuritis, papilledema, and acute cranial nerve (CN) paresis, including abducens and oculomotor nerve involvement, have been reported^(2-8)^. Isolated abducens nerve palsy is the most common CN palsy and can result from various etiologies^(4)^. This report is unique in different respects, as we describe a 48-year-old male patient who presented isolated abducens nerve palsy after COVID-19, with no evidence of CN involvement on neuroimaging and no other neurological or vascular abnormalities. In addition, we describe the complete and spontaneous improvement of the case over a period of 8 months.

## CASE REPORT

A previously healthy 48-year-old man presented to the Hospital de Olhos de Pernambuco, Recife, Brazil, with acute diplopia. The patient’s past medical history revealed clinical manifestations of SARS-CoV-2 infection (e.g., fever, fatigue, headache, ageusia, and anosmia) 18 days prior to the ocular complaint. Dyspnea persisted for 1 day, but the chest computed tomography (CT) finding was normal. The COVID-19 diagnosis was confirmed by laboratory examinations, with reverse transcriptase polymerase chain reaction (RT-PCR) for SARS-CoV-2. The patient received oral azithromycin and ivermectin and was not hospitalized. One day prior to presenting diplopia, the patient took a pill of cyclobenzaprine hydrochloride (10-mg Miosan), a muscle relaxant, because he experienced headache and body pain.

**Figure 1:**
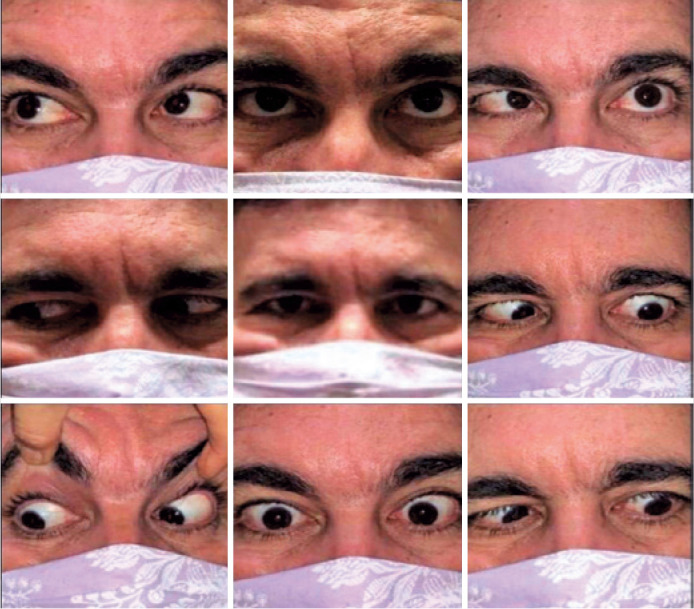
Left abducens nerve palsy in a previously health male patient after coronavirus disease.

In the initial ophthalmologic examination, a complete ophthalmologic examination was performed and revealed a best-corrected visual acuity of 20/20 in both eyes (oculus uterque [OU]), an esotropia (ET) of 30 PD, and no stereoacuity. Abduction limitation (-3) was observed in the left eye (oculus sinister [OS]; Figure 1). Pupillary reactions were normal bilaterally, and no nystagmus was observed. The patient presented an abnormal head posture, turning the face to the left to compensate for the horizontal misalignment and diplopia. Intraocular pressures, anterior biomicroscopy, and fundoscopy were normal in OU.

Additional ancillary ophthalmologic examinations, including visual field, pattern electroretinography, opti cal coherence tomography, and color fundus photography were performed and revealed normal findings.

The patient was referred for a complete workup, including clinical, cardiological, neurological, neuroimaging (brain magnetic resonance imaging [MRI]), blood, and cerebrospinal fluid sample analysis. Other systemic and infectious disease causes were ruled out.

The patient opted for a noninvasive and conservative approach. He self-initiated orthoptic therapy and eye patching of the right eye for a few hours each day. Three months after the onset of COVID-19, he presented partial remission of the sixth CN palsy. The ET was reduced to 15 PD, and the E(T)′ was 6 PD. The ophthalmologic examination also revealed an improvement of the head posture, binocular vision, and stereopsis. Fresnel prism was prescribed for the deviating eye to eliminate diplopia.

**Figure 2:**
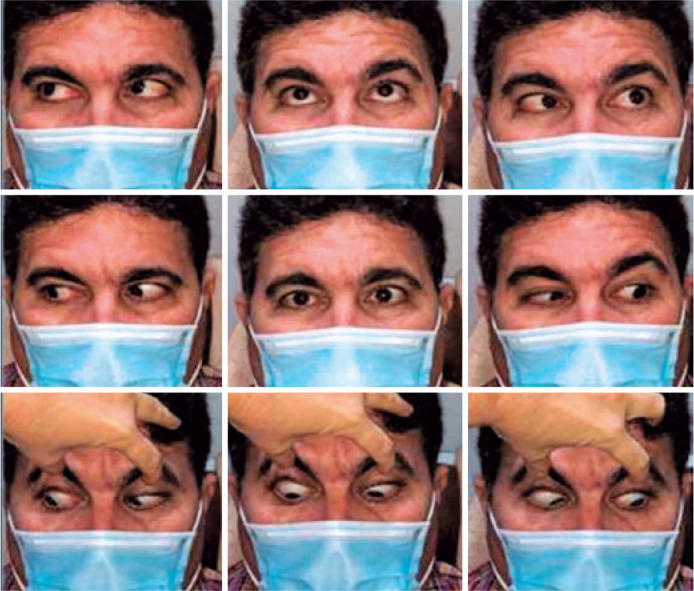
Three-month follow-up of a left abducens nerve palsy in a previously health male patient after coronavirus disease.

Progressive follow-up showed resolution of the sixth CN palsy without associated neurological signs or symptoms (Figure 2). Eight months later, the patient reported complete recovery of the diplopia.

## DISCUSSION

Acquired CN palsies in adults are usually associated with microvascular diseases, and causes include vasculopathies, tumors, and inflammatory conditions^(3,8)^. The potential association of SARS-CoV-2 infection with CN involvement has been previously described, and its association with diplopia was recently reported^(3-8)^. Most cases were described in previously healthy young adult men, while elderly women were more affected^(4-8)^. The most affected CN was the abducens, followed by the oculomotor nerve^(4-8)^. Our case supports previous reports, as the patient presented with isolated abducens nerve palsy 18 days after presenting COVID-19 symptoms and having laboratory confirmation with RT-PCR.

Studies have shown that neurotropic viruses, including alpha herpesviruses, varicella zoster, and chikungunya, may cause craniofacial palsies^(4,9)^. Management of these cases include treatment of the baseline disease in combination with a conservative approach for the diplopia, including unilateral eye occlusion and Fresnel prisms, as opted in this case report^(9)^. When ocular motility is not recovered after 6 to 12 months, strabismus surgery may be considered^(9)^. In the present case, the strabismus and diplopia resolved within 8 months.

In contrast to previous cases, our patient showed a mild clinical presentation of COVID-19, did not present any perineural or cranial findings on CT/brain MRI, and did not need hospitalization^(4-8)^. This highlights the wide spectrum of clinical, neurological, and neuro-ophthalmologic manifestations of COVID-19.

With regard to the pathophysiology of the neurological complications related to SARS-CoV-2 infection, much remains unknown. Vonck et al. showed that SARS-CoV-2 uses angiotensin-converting enzyme 2 (ACE2) to invade human cells. As ACE2 receptors are present in the glial cells and neurons, they concluded that part of the neurological impairment in COVID-19 may be due to direct viral neurological injury or indirect autoimmune and neuroinflammatory mechanisms^(2-3)^. Thus, the abducens nerve involvement in this case could be due to a direct or indirect insult of the nerve caused by the SARS-CoV-2 infection, as suggested by Falcone et al.^(4)^.

As far as we know, this is the first report to describe an 8-month follow-up of a patient with sixth CN palsy secondary to COVID-19. Owing to the lack of information on this novel entity and its wide range of complications, the recognition of acquired sixth CN palsy secondary to COVID-19 by ophthalmologists is important for the proper diagnosis and management of patients. We suggest a more conservative approach with orthoptic therapy, ocular patching, and Fresnel prisms before considering more invasive therapies such as botulinum toxin or other surgical approaches for these cases.
